# Cardiac surgeons between apprehension and ethical duty in the COVID-19 pandemic

**DOI:** 10.1177/0218492320943355

**Published:** 2022-10

**Authors:** Ahmed MA Bakry, Ehab Sobhy, Hysam Abdelmohty

**Affiliations:** 1 Cardiothoracic Surgery Department, Zagazig University, Egypt; 2 Cardiothoracic Surgery Department, Mansoura University, Egypt

**Keywords:** Cardiac surgical procedures, coronavirus infections, Egypt, operating rooms, thoracic surgical procedures

## Abstract

**Background:**

Cardiothoracic surgeons are facing a big challenge in their surgical practice in the era of the COVID-19 pandemic. The attitude towards performing surgery is influenced by the pandemic. Setting special recommendations for safe cardiothoracic surgery is of extreme importance.

**Methods:**

This was an observational cross-sectional survey that included 77 Egyptian cardiothoracic surgeons. The survey consisted of a self-administered constructed questionnaire with six sections, and was delivered as a Google Forms questionnaire (https://www.google.com/forms/about) that was sent to individuals via social networks and email.

**Results:**

More than 80% of Egyptian cardiothoracic surgeons believe they and their patients are at risk. Out of all participants, none had actually been infected with COVID-19 but 26% had encountered a positive COVID-19 person in their surgical team. Although 51% were testing patients before surgery, they reported 9 confirmed cases postoperatively. Computed tomography was the most recommended investigation prior to surgery (by 69%). Most had postponed elective surgeries and only one-third of all surgeons recommended performing elective surgeries cautiously with pretesting for COVID-19 and maximizing protective measures, while more than 40% recommended not performing high-risk elective surgeries.

**Conclusion:**

We are committed to the safety of our patients, ourselves, our staff, and our families. Planning for the new phase of reopening, whether total reopening or step-by-step reopening, should carefully consider how we should utilize our resources, respect social distancing, and prevent exposure to untested patients or health workers who might turn out to be an undetected positive case.

## Introduction

Since the discovery of the first case in a fish market in Wuhan, China, in December 2019,^1^ there has been worldwide focus on COVID-19. Meanwhile, the patient load trajectory is exceeding the capacity of any health system, occupying all intensive care unit (ICU) beds and ventilators. In addition, the world is suffering from an unprecedented universal shortage of protective materials for frontline health professionals, including masks and gloves. There is also a shortage of healthcare workers agreeing to work under the pandemic, and there are newly sick healthcare staff such as nurses and paramedics who came in direct contact with untested patients. The identification of COVID-19 cases is guided by criteria constantly updated by the Egyptian Ministry of Health in accordance with World Health Organization (WHO). The COVID-19 average incubation period ranges from 5.2 days up to 14 days. The average period of transmission of this disease is 5 days after the onset of symptoms. Unfortunately, 10% of those infected are expected to be severe cases, and 5% will require admission to an ICU.^1,2^ The risk of catching coronavirus infection by patients undergoing cardiothoracic surgery and developing COVID-19 during or after cardiac surgery is quite high. The presence of heart disease itself is a risk factor for developing a severe form of COVID-19, and the risk becomes higher in patients with associated comorbidities.^3^ The prolonged hospital stay after these major surgeries represents an additional risk. Moreover, COVID-19 has been associated with cardiac insult. An elevated ultrasensitive troponin I was found in more than half of the deaths, contributing to the findings of acute cardiac injury in up to 12% of COVID-19 cases.^3^ Cardiothoracic surgeons are facing big challenges in their surgical practice in the era of the COVID-19 pandemic. The attitude of cardiothoracic surgeons towards performing surgery may be influenced by this pandemic. Setting special recommendations for safe cardiothoracic surgery during the COVID-19 pandemic is of extreme importance. This is the first study to target Egyptian cardiothoracic surgeons, reporting the impact of the COVID-19 pandemic on their attitude and behavior. We collected and evaluated their fears and recommendations for safe surgical practice in the presence of COVID-19.

## Methods

This was an observational cross-sectional survey that included Egyptian cardiothoracic surgeons. Sample size was calculated based on the total of 650 registered with the Egyptian Society of Cardiothoracic Surgery, with a prevalence of the factor under study of 94% and a 95% confidence level. The sample size was calculated to be 77 cardiothoracic surgeons. The study was approved by the Institutional Review Board in Zagazig University Hospitals, in accordance with the code of ethics of the World Medical Association (Declaration of Helsinki) for studies involving humans.

For sample collection, given the quarantine situation forced by the COVID-19 outbreak, the questionnaire was delivered as a Google Forms questionnaire (https://www.google.com/forms/about) that was sent to individuals via social networks and emails. Exclusion criteria included non-Egyptian surgeons and questionnaires with fewer than 50% of the items completed.

The survey used in this study consisted of a self-administered constructed questionnaire that included six sections: an introduction to the survey, personal and practice setting information (7 questions), attitude (8 questions), behavior (8 questions), COVID-19 infection encountered in the surgical team or patients (5 questions), and recommendations regarding surgical practice (8 questions).

Data were collected from Google Forms on an Excel sheet, recoded, entered, and analyzed using Statistical Package for Social Science version 14 (SPSS, Inc., Chicago, IL, USA). Age is presented as mean and standard deviation. Cardiothoracic surgeons’ responses are presented as number and percentage, and some figures were derived from Google Forms.

## Results

**Figure 1.**
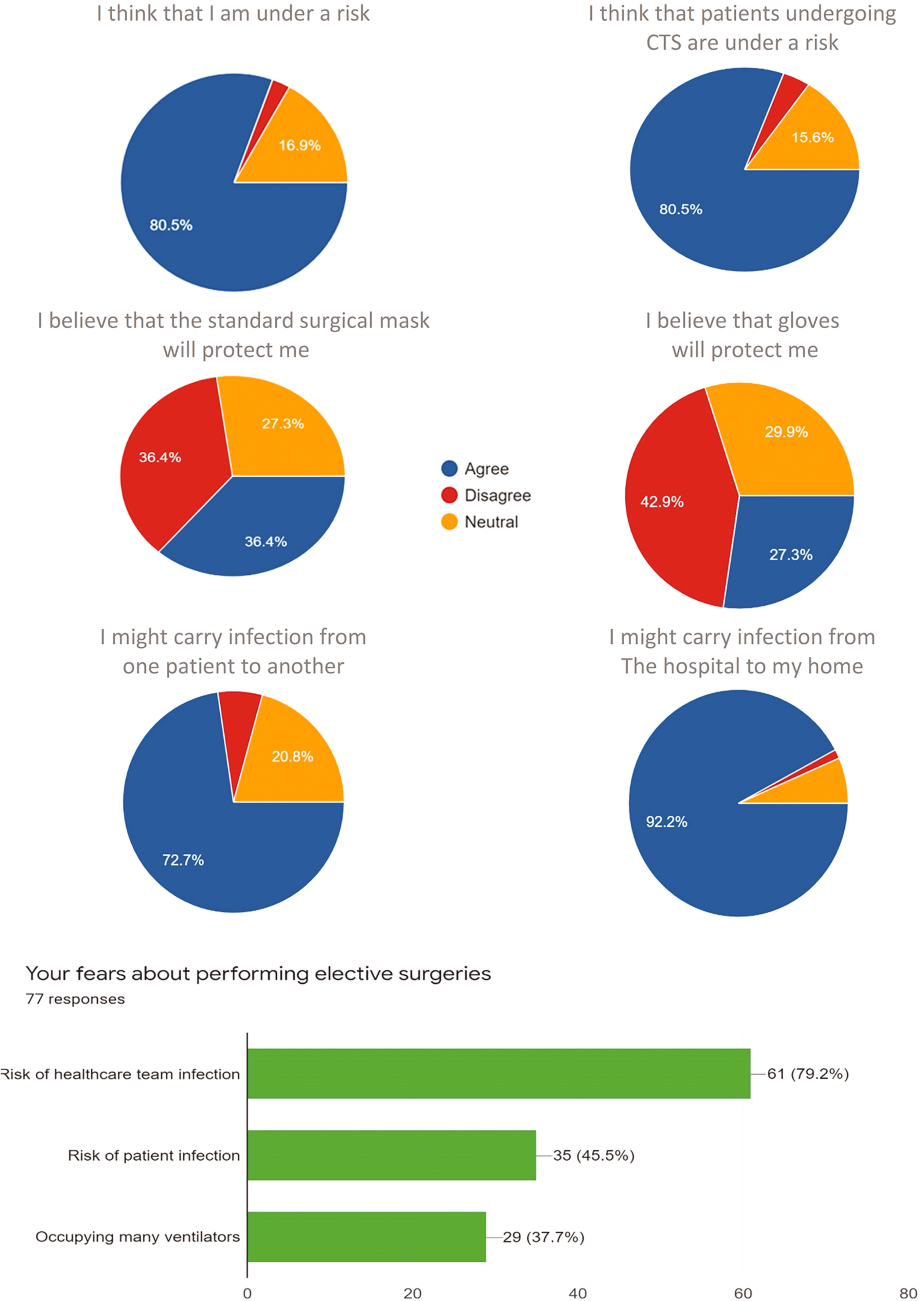
The attitude of cardiothoracic surgeons regarding risk of infection with coronavirus. CTS: cardiothoracic surgery.

**Figure 2.**
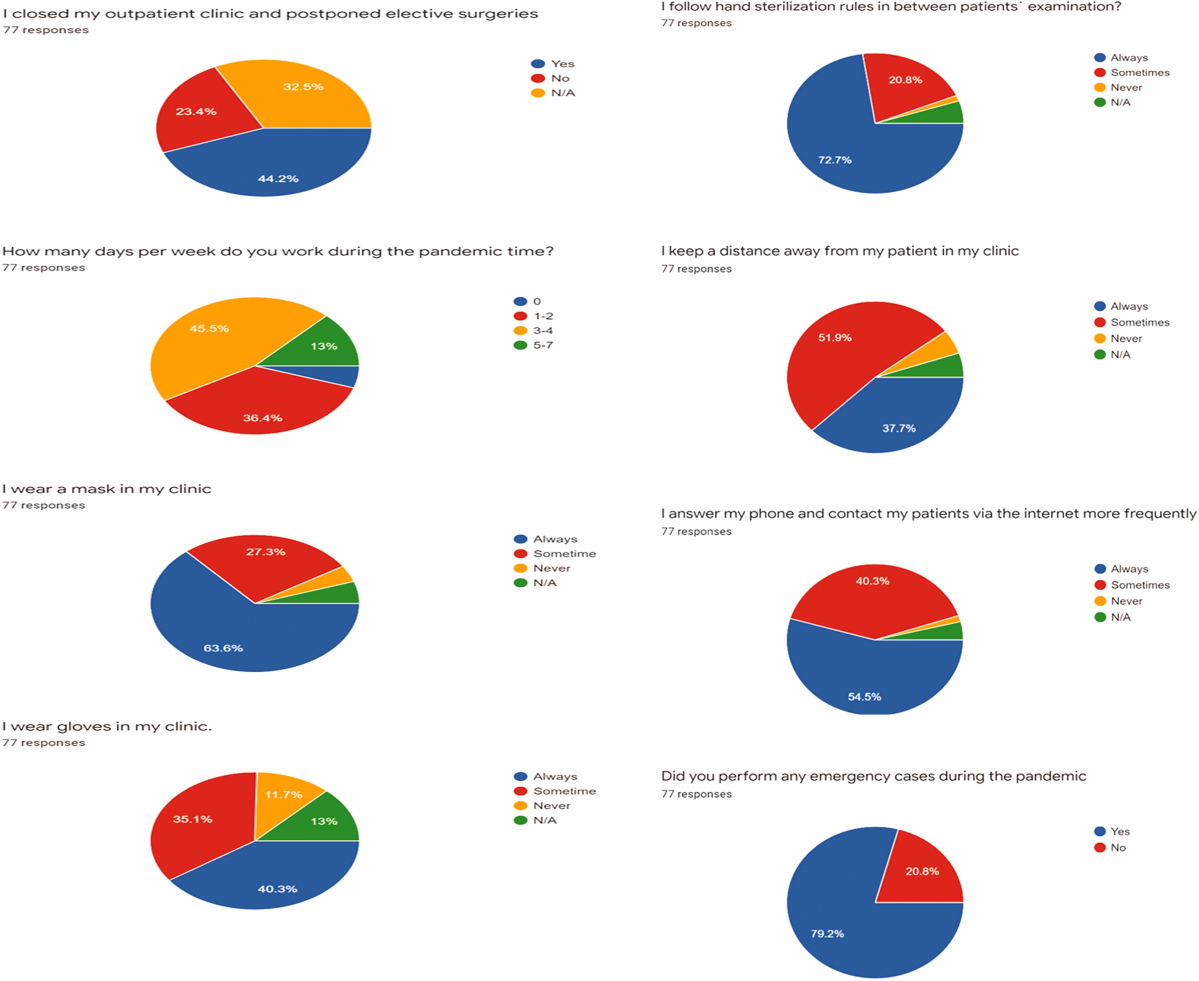
Behavior of cardiothoracic surgeons in clinical practice.

**Figure 3.**
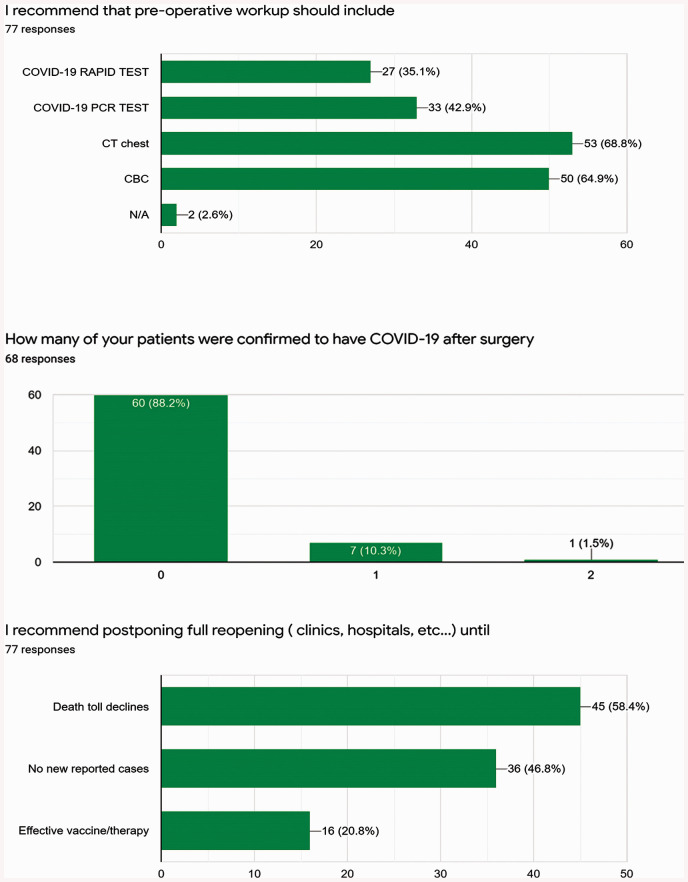
Number of patients confirmed to have COVID-19 after surgery and recommendations of cardiothoracic surgeons regarding pretesting and reopening hospitals and clinics.

Table 1 shows the data of cardiothoracic surgeons involved in this study. The majority were consultants, smokers, and had a preexisting medical condition. Regarding their attitude, more than 80% agreed that they are at risk and this was their major fear, as shown in Figure 1. More than 90% agreed that they may carry the infection home. They also believed that their patients are at risk and that they themselves may carry infection from one patient to another. Results regarding their behavior are shown in Figure 2. Just under 80% had actually carried out emergency surgery since the start of the pandemic. The majority contacted their patients via telephone or internet for follow-up more often than usual. Nearly a half of all cardiothoracic surgeons participating worked 3 to 4 days per week during the pandemic. Many used masks in their clinics but fewer used gloves, however, more than 70% used hand sanitizers between patient examinations. None had actually been infected with COVID-19 but 26% stated that they had encountered a COVID-19-positive person in their surgical team. Although 51% were testing patients before surgery, they reported 9 cases confirmed to have COVID-19 postoperatively. These cases developed postoperative fever, cough, pneumonia, prolonged ventilation, and even respiratory failure. They recommended a preoperative workup that included a COVID-19 rapid test, a COVID-19 polymerase chain reaction test, a complete blood count, and serum ferritin (Figure 3 and Table 2). Computed tomography (CT), recommended by 69%, was the most recommended investigation prior to surgery. One-third of all surgeons recommended performing elective surgery cautiously with pretesting for COVID-19 and maximizing protective measures, while more than 40% recommended against performing high-risk elective surgeries. They also recommended increasing the availability of personal protective equipment for the entire surgical team, raising the level of suspicion, and performing CT in all patients preoperatively. They further recommended the development of a COVID-19 crisis management team in each cardiac surgery department. Regarding reopening, approximately 80% recommended step-by-step reopening. Full reopening of hospitals and clinics was advised by many to be postponed, based mainly on newly reported cases and the death toll. All recommendations are shown in Table 2.

**Table 1.** Personal data and practice setting of participating cardiothoracic surgeons.

Variable	No. of patients
Age (years)	41.9 + 10.3
Male	75 (97.4%)
Female	2 (2.6%)
Job title	
Consultant	41 (53.2%)
Specialist	27 (35.1%)
Resident	9 (11.7%)
Are you a smoker?	
Yes	66 (85.7%
No	11 (14.3%)
Do you have a preexisting medical problem?	
Yes	67 (87%)
No	10 (13%)
Your practice setting?	
University hospital	39 (50.6%)
Public hospital	32 (41.6%)
Private hospital	6 (7.8%)
Does your work place provideprotective equipment?	
Yes	44 (57.1%)
No	33 (42.9%)

**Table 2.** Recommendations of Egyptian Cardiothoracic Surgeons.

Recommendation	Agree	Disagree	Neutral
Total closure of outpatient clinics	18 (23.4%)	37 (48.1%)	22(28.6%)
Postpone all elective surgeries	33 (42.9%)	27 (35.1%)	17 (22.1%)
Perform elective surgery cautiously, pretesting for COVID-19			
and maximizing protective measures	49 (36.6%)	19 (24.7%)	9 (11.7%)
No high-risk elective surgery	34 (44.2%)	27 (35.1%)	16 (20.8%)
Step-by-step reopening in the meantime	62 (80.5%)	6 (7.8%)	9 (11.7%)
Additional recommendations			
Increased availability of protective equipment for all surgical teams			
Perform antibody tests + computed tomography in all patients			
All trauma patients to have computed tomography on admission			
COVD-19 crisis management team in each cardiac surgery department			
Serum ferritin level prior to surgery			

## Discussion

Fighting for survival during the COVID-19 pandemic, it is essential to start recording our observations on cardiothoracic surgical practice, paving the way for a more in-depth understanding of the new challenges forced during the pandemic. Pandemics such as respiratory H1N1 influenza, Spanish flu, Swine flu, and lately, COVID-19, brought humanity together and taught us lessons in coherence and solidarity. However, eventually, early social distancing was the greatest lesson learnt from the fights against previous viruses that swept the globe over the last few decades. The apprehension and concerns of cardiac surgeons regarding silent coronavirus carriers is acceptable; “Without having a properly organized working environment, operating room and ICU, how we can ensure our safety and patients’ safety before us!”

Cardiothoracic surgeons play an important role in reinforcing good healthcare practices in their clinics, units, and community, starting with washing hands with soap and water, avoiding touching the face with the hands, not sharing personal items, using tissues to sneeze or cough, rubbing alcohol gel on the hands between patient examinations, keeping a distance from patients, wearing protective masks and gloves in clinics, limiting the number of patients seen each day, performing urgent surgery under protective precautions, limiting the time of surgery, and limiting the number of attending personnel. For the sake of preservation of the manpower running the unit, it is better to distribute work days/weeks among 4 or more surgeons/professional groups, so if they are exposed to positive cases, isolation can be applied to the exposed group only within each unit, rather than the entire unit with the possible closure of the entire service. Nearly half of all cardiothoracic surgeons participating in this survey worked 3 to 4 days per week. Many of them used masks in their clinics but fewer used gloves, however, more than 70% used hand sanitizers between patient examinations.

COVID-19 is not airborne and it spreads principally by droplets. Therefore, the use of gowns, gloves, an N95 respirator plus a face shield and/or googles when treating patients with COVID-19 is mandatory and effective.^4^ No clinical data are available on whether an N95 mask gives greater protection than a regular surgical mask to the surgical team. However, because techniques such as tracheal intubation, bronchoscopy, and esophagoscopy can generate aerosols of virus-containing particles that can be inhaled, N95 masks plus face shields should be recommended. This becomes necessity if the surgical candidate is either an asymptomatic carrier or a COVID-19 patient. Among our surgeons, only one-third believed that the standard surgical mask would protect them.

No guidelines for COVID-19 screening in patients requiring cardiac surgery have emerged. The fact that either the patients requiring surgery or the healthcare staff might be asymptomatic carriers of the virus makes us willing to perform COVID-19 screening as a primary requirement before surgery. Understanding that screening results come 2–3 days later, it is unreasonable that patients needing cardiac surgery during the COVID-19 pandemic be screened for the virus then sent back home for the next 3 days where they might catch the infection while waiting for the results. In our survey, despite the fact that more than half of our Egyptian cardiothoracic surgeons were already testing their patients before surgery, they reported that 9 patients developed COVID-19 after surgery. CT was the most recommended investigation by the participants, due to the characteristic appearance of COVID-19 infection. In addition, a complete blood count, serum ferritin, and COVID-19 testing were also recommended. If we have the resources available, cardiac surgery should be only performed in an operating room with a negative-pressure environment to reduce dissemination of the virus. If a negative-pressure room is not available, a greater frequency of air exchange will help to reduce the viral load within the operating room. Operating room doors should remain closed throughout the procedure. The number of attending persons should be reduced, and all healthcare workers must wear full personal protective gear while caring for these patients. Single-use instruments should be discarded after surgery in a sealed container, while others must be impregnated in disinfectant solution and sterilized. Meticulous cleaning of surfaces with disinfectant before and after the procedure is mandatory.^5^

Symptoms of COVID-19 may be mild, and it can present with heart palpations and chest tightness, rather than with respiratory symptoms such as fever and cough, so its diagnosis in healthcare workers can be difficult.^2^ Those with symptoms of COVID-19 should not continue to provide direct patient care and should be tested for COVID-19. They should be separated in an isolation room. Whether or not they should continue working using surgical masks until the test results are available is a matter of debate, but theoretically, they should not. If positive for COVID-19, healthcare workers must receive medical treatment and quarantine themselves immediately. Those negative should self-isolate for a couple of weeks before returning to duty. The problem is that these recommendations may lead to a shortage of healthcare staff. Moreover, the number of qualified cardiothoracic surgeons in Egypt is limited because this specialty has a long learning curve and requires many years of training, so they should be optimally protected. Fortunately, none of the surgeons participating in the survey had actually been infected with COVID-19 but 26% stated that they had encountered a positive COVID-19 person in their surgical team.

Anticipating the inevitable demand for ICU beds and the possible need for every ventilator for COVID-19 patients has urged many European countries to indefinitely cancel all elective surgery. A reality foreseen, the Egyptian Ministry of Health recommended postponement of elective surgery nationwide. One bright face of the crisis is working from home using telemedicine. Telemedicine and teleconferences swept the healthcare field with emerging mobile apps. There has been an expansion of private medical consultation services that have changed the surgeon-patient relationship.^6^ Many of our cardiothoracic surgeons contacted their patients via telephone or internet for follow-up more often than usual. Of all participants in the survey, approximately 44% actually postponed their elective surgeries. Only one-third of all surgeons recommended performing elective surgery cautiously, pretesting for COVID-19 and maximizing protective measures, however, more than 40% recommended against performing high-risk elective surgeries. Patients in advanced age groups usually suffer 2 or more comorbidities and tend to stay for longer periods in hospital after cardiac surgery.^7^ The fatality of COVID-19 is higher if patients are over 50-years old, with rates ranging between 1.3% and 14.8% in the over 80-years age group.^8^

The recommendations of the United Kingdom’s National Health Service, the Society for Cardiothoracic Surgery in Great Britain and Ireland, and the American College of Surgeons did not state which patients should be operated on, and did not recommend pretesting for COVID-19.^9,10^ Meanwhile, in Italy, the Maria Cecilia Hospital in Cotignola, Ravenna, is screening every patient with a swab test for COVID-19. In Egypt, approximately 80% of Egyptian cardiothoracic surgeons have treated emergency cases since the start of the pandemic. Cardiac surgeons have operated on emergency cases of acute aortic dissection, stuck mechanical valves, tight left main disease, and cardiac trauma. Moreover, some severe elective cases have been operated on, such as patients with severely symptomatic coronary artery disease and severe valvular pathologies with impaired function.

The COVID-19 pandemic is changing into an endemic. That essentially will mean changes in the social and health services responses and tactics towards the threat. The phase of early detection and containment of focal cases, and banning travelers from entering infected areas, aimed to prevent more cases and eventually reduce deaths. The next phase of reopening is expected with speculation and apprehension. Regarding reopening, approximately 80% of our surgeons recommended step-by-step reopening. Full reopening of hospitals and clinics was advised by many to be postponed, based mainly on newly reported cases and the death toll.

We are committed to the safety of our patients, ourselves, our staff and our families. Planning for the new phase of reopening through the few months ahead, whether total reopening or step-by-step reopening, should carefully consider how we should utilize our resources, respect social distancing, and prevent exposure to un-tested patients or healthcare workers who might turn out to be undetected COVID-19-positive. We need to work together towards implementing many operational changes across our healthcare system and modifying our criteria for performing invasive procedures. We should be taking these protective steps especially at this time because of the documented accelerating community spread of COVID-19.
